# Congenital and hereditary cystic diseases of the abdomen

**DOI:** 10.1186/s13244-020-00898-z

**Published:** 2020-08-05

**Authors:** Ali Devrim Karaosmanoglu, Sevtap Arslan, Deniz Akata, Mustafa Ozmen, Mithat Haliloglu, Berna Oguz, Musturay Karcaaltincaba

**Affiliations:** Department of Radiology, Hacettepe University School of Medicine, 06100 Ankara, Turkey

**Keywords:** Abdomen, Cystic lesions, Hereditary, Congenital

## Abstract

Congenital and hereditary cystic lesions of the abdomen are relatively rare. Correct diagnosis is critical as they may simulate several other benign and malignant acquired diseases of the abdomen. With the correct and appropriate use of imaging, diagnosis may be relatively straightforward and clinical management may be implemented appropriately. The purpose of this article is to describe imaging findings of common and uncommon congenital and hereditary cystic disease of the abdominal organs.

## Key Points

- The detection of incidental cystic lesions may cause diagnostic confusion in patients having a history of cancer.
- Some syndromes predisposing the affected individual to different tumors may cause cystic lesions in the abdominal organs and rarely, the tumors can be cystic.
- Correct diagnosis of the benign cystic lesions is critical as they may simulate several other benign and malignant acquired diseases of the abdomen, all of which have very different treatment approaches and prognostic implications.

## Introduction

Abdominal cystic lesions may originate from parenchymatous organs or from nonparenchymatous structures and may be congenital, hereditary, or acquired. The organ of origin, the position of the cystic lesion, and specific imaging findings are useful in the differential diagnosis. Ultrasonography (US), computed tomography (CT), and magnetic resonance imaging (MRI) may be used for imaging, all of which have pros and cons. The US, which is widely available and less expensive, can be preferable in pediatric patients because of the lack of ionizing radiation and the opportunity of real-time imaging but one of its main disadvantages is operator dependence. CT and MRI may also be used for assessment of the anatomical relations and, in addition, the internal content of these cystic structures may also be effectively evaluated with these modalities. They can be used for follow-up purposes for lesions with a potential risk of malignant transformation. One of the main advantages of CT as compared to MRI is its allowance of rapid image acquisition, especially in non-cooperative patients. MRI, which provides high soft-tissue resolution, might be preferable for follow-up, especially in young patients, due to lack of ionizing radiation. Knowledge of the typical imaging findings for cystic diseases can help radiologists in establishing the correct diagnosis. So, the radiologist can provide valuable information to the clinician to guide further management. In this review, we describe imaging findings of the congenital and hereditary cystic diseases of the abdomen. A detailed literature search was also carried out to be able to summarize the radiological findings of these particular cystic diseases (Table 1) [1–33].

**Table 1:** Congenital and hereditary cystic diseases of the abdomen. Associated radiological findings.

Organ	Disease		Typical imaging findings
**Liver and biliary tract** *[1–9]	Polycystic liver disease		- Cysts located within the peripheral parenchyma - Peribiliary cysts - Fluid level, cyst wall thickening, calcification and endocavitary air bubbles, if infection is present - Hyperdensity on CT and hyperintensity on T1W MR images could be seen due to the hemorrhage or infection
	Caroli disease		- Cystic appearing enlarged intrahepatic bile ducts - Central dot sign (portal radicle within the dilated bile duct) - Endoluminal stones or sludge may be observed
	Choledochal cysts	Type-I	- Type IA: Diffuse cystic dilation of the extrahepatic bile duct - Type IB: Focal cystic dilation of the extrahepatic bile duct - Type IC: Diffuse fusiform dilation of the entire extrahepatic bile duct
		Type-II	- Focal diverticular outpouching of the common bile duct
		Type-III	- Intramural dilation of the most distal portion of the common bile duct (choledochocele)
		Type-IV	- Type IVA: Combined saccular shaped dilations in the intrahepatic and extrahepatic bile ducts - Type IVB: Saccular dilations restricted to extrahepatic bile ducts
		Type-V	- Caroli disease
	Biliary hamartomas(von Meyenburg complex)		- Innumerable subcentimeter cysts spread throughout the liver parenchyma
	Ciliated hepatic foregut cyst		- Unilocular cystic lesion located in the subcapsular area along the anterior surface of the liver with segment 4 being the most common location
**Kidney** *[10–19]	Autosomal dominant polycystic kidney disease		- Early stage: Single or multiple cysts in one or both kidneys - Final stage: Multiple cysts completely replacing the entire renal parenchyma - Hyperdensity on CT ^(a)^ and hyperintensity on T1W^(b)^ MR^(c)^ images in case of hemorrhage
	Autosomal recessive polycystic kidney disease		- Enlarged kidneys with thickened hyperechoic parenchyma caused by microcysts - Larger cysts (>1 cm) may accompany in some cases - Suggestive findings of hepatic abnormalities including congenital hepatic fibrosis, Caroli disease, and bile duct ectasia
	Multicystic dysplastic kidney disease		- Unilateral cysts in disorganized pattern completely replacing the renal parenchyma, which may be observed on antenatal US^(d)^
	Nephronophthisis and medullary cystic kidney disease		- Early stage: Hyperechoic renal parenchyma with the loss of corticomedullary differentiation - Advanced stage: Cysts, of varying size, in medullary and corticomedullary locations. The kidneys appear small due to parenchymal fibrosis
	Von Hippel-Lindau disease		- Bilateral renal cysts of varying histopathologic features, ranging from simple and hyperplastic cysts to cystic clear cell carcinomas
	Tuberous sclerosis complex		- Bilateral simple renal cysts with accompanying angiomyolipomas
**Pancreas** *[20–23]	Von Hippel-Lindau disease		- Simple cysts - Serous cystadenomas - Cystic or solid neuroendocrine tumors
	Multiple endocrine neoplasia type I		- Cystic or solid neuroendocrine tumors
	Cystic fibrosis		- Complete or partial fatty replacement of the pancreas - Atrophy of the pancreas - Simple cysts completely replacing the parenchyma (pancreatic cystosis)
**Gastrointestinal tract** *[24–26]	Duplication cysts		- Cyst within the close proximity of the bowel segment - The double wall sign (inner hyperechoic mucosa and outer hypoechoic muscularis propria) - “Y configuration” that is indicative of a shared wall with the cyst and the neighboring bowel wall - Internal septation or luminal debris may be observed due to the infection
**Lymphatic system** *[27, 28]	Lymphatic malformations		- Well-circumscribed cystic lesion with internal septations - The fluid content of the lesion may contain fat - Small lesions may change location on follow-up imaging
**Diaphragm** *[29]	Mesothelial cyst		- Homogeneous bilobulated cystic lesion located between posterolateral aspect of the right liver lobe and the adjacent diaphragm
**Prostate** *[30, 31]	Prostatic utricle cyst		- Midline cyst communicating with the prostatic urethra and not extending above the base of the prostate
	Mullerian duct cyst		- Teardrop-shaped midline cyst extending above the superior margin of the prostate and not communicating with the prostatic urethra
**Urachus** *[32]	Urachal cyst		- Homogeneous midline cyst along the trajectory of the urachus (between the bladder dome and umbilicus) - Inhomogeneous cyst content, cyst wall thickening, and inflammatory stranding adjacent to the cyst indicate infection
**Zinner’s syndrome** *[33]	Seminal vesicle cysts		- Ipsilateral renal agenesis, seminal vesicle cysts, and ejaculatory duct obstruction - In case of hemorrhage or infection the cyst content may appear as bright on T1W MR images

*: References , ^(a)^ CT: Computed tomography, ^(b)^ T1W: T1-weighted, ^(c)^ MR: Magnetic resonance, ^(d)^ US: Ultrasonography

## Liver and biliary tract

### Polycystic liver disease

Polycystic liver disease (PLD) is a part of the spectrum of fibropolycystic liver disease. It has an autosomal dominant inheritance pattern and may also be related to polycystic kidney disease (PKD). This association with PKD is not rare and may be seen in around 50% of the patients [34]. It is a rare disease, with an estimated incidence of < 0.01%, with a slight female preponderance [34, 35]. Genetic mechanisms are the most important underlying cause which gives rise to the separation of ductal structures from the biliary tree, ultimately resulting in cyst formation [36]. These disconnected bile ducts typically remain clinically silent until cysts begin to form in adulthood [36]. These cysts are not distinct from simple hepatic cysts from a histopathologic standpoint. Their walls are lined by cuboidal biliary epithelium and contain serous fluid in their cavities [1]. The cysts tend to emerge after puberty and they generally remain asymptomatic. Cyst rupture, hemorrhage, or infection may be counted among the potential complications of these cysts. Malignant degeneration and liver failure are rare. Liver transplantation is reserved for symptomatic relief [37].

**Fig. 1:**
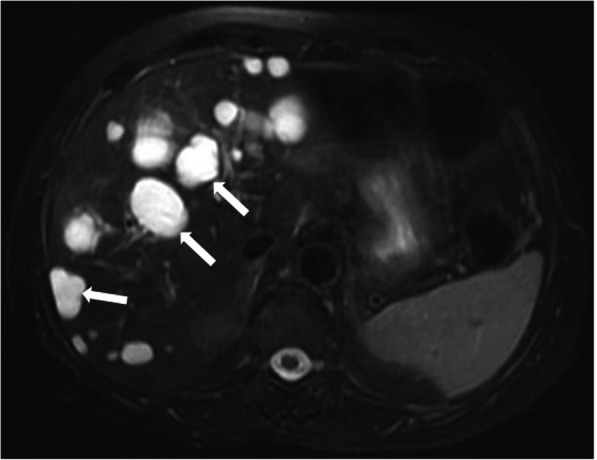
A 60-year-old female patient with known long-standing PLD. Axial plane T2W fat-saturated MR image shows multiple parenchymal cysts in the liver (arrows)

**Fig. 2:**
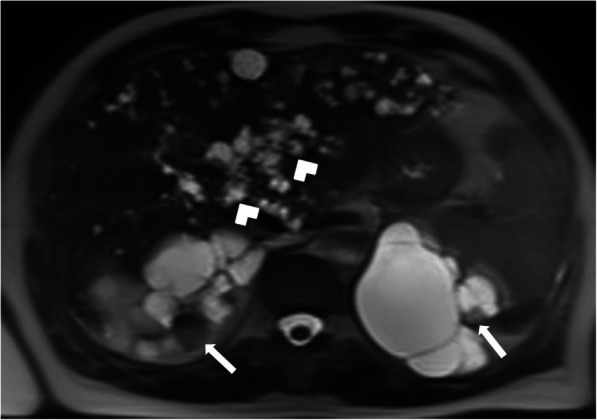
A 55-year-old female patient with known ADPKD and PLD. Axial plane T2W fat-saturated MR image shows multiple bilateral renal cysts with hemorrhagic cysts (arrows) and peribiliary cysts (arrowheads) in the liver

**Fig. 3:**
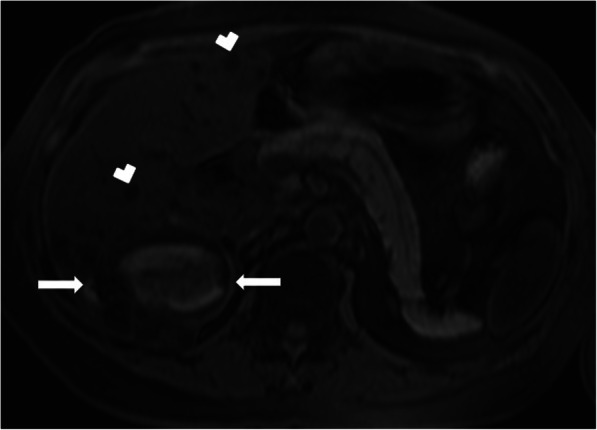
A 44-year-old female patient with known PLD presenting with right upper quadrant pain. On the US, a large hyperechoic mass was found in the right hepatic lobe (not shown). Axial plane precontrast T1W MR image shows subcentimeter cysts (arrowheads) and a cystic lesion with hyperintense content (arrows). There was no discernible enhancement on postcontrast series (not shown). Imaging findings were found to be consistent with the hemorrhagic cyst. Follow-up imaging studies demonstrated the decrease in the size of the lesion

**Fig. 4:**
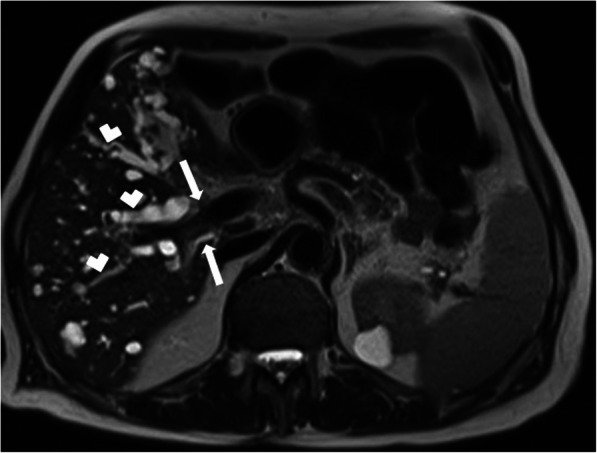
A 60-year-old male patient with known PLD presenting with recent onset jaundice. Axial plane T2W MR image demonstrates innumerable cysts scattered throughout the liver parenchyma. Also, note is made of dilation in the intrahepatic bile ducts (arrowheads). This dilation abruptly ends at the confluence of the right and left hepatic ducts (arrow). The patient underwent endoscopic retrograde cholangiopancreatography and endoscopic brush biopsy confirmed Klatskin tumor.

On cross-sectional imaging, the cysts are typically located within the peripheral parenchyma (Fig. 1). They highly vary in size, ranging from a few millimeters to 80 mm [1]. The peribiliary cysts, in the periportal distribution, may also be seen and they are typically small (< 10 mm) (Fig. 2). The cysts tend to increase both in size and number with the advancing age. Infected or hemorrhagic cysts may appear as hyperdense on CT and hyperintense on T1-weighted (T1W) MR images [2] (Fig. 3). Fluid level, cyst wall thickening, calcification, or endocavitary air bubbles may be seen in infected cysts [3]. Non-complicated cysts have well-defined margins and the cyst walls are smooth without any mural nodularity. Considering the LI-RADS v2018 (Liver Imaging Reporting and Data System Version 2018), non-complicated cysts are categorized as LR-1 lesions, which also include typical hemangiomas, vascular anomaly, confluent fibrosis, hepatic fat deposition or sparing, and focal scar [38]. Hepatocyte-specific contrast agents are useful for demonstrating the absence of any communication between the biliary system and the cysts [2]. Although it is rare, the possibility of concomitant cholangiocarcinoma should be considered in patients with PLD and abnormal liver function tests [39] (Fig. 4).

### Caroli disease

Caroli disease (CD) manifests with saccular, non-obstructive, multisegmental dilation of the large intrahepatic bile ducts [4]. The disease is mostly inherited in an autosomal recessive fashion. In the so-called pure form of CD, there is no associating background parenchymal liver abnormality. In the coexistence of CD and congenital hepatic fibrosis, the disease process is called as the “Caroli syndrome”, which is the more commonly encountered disease form. CD is also included in the Todani classification and classified as type V abnormality [4]. The disease mostly manifests around 30 years of age; however, patients with Caroli syndrome may present earlier.

**Fig. 5:**
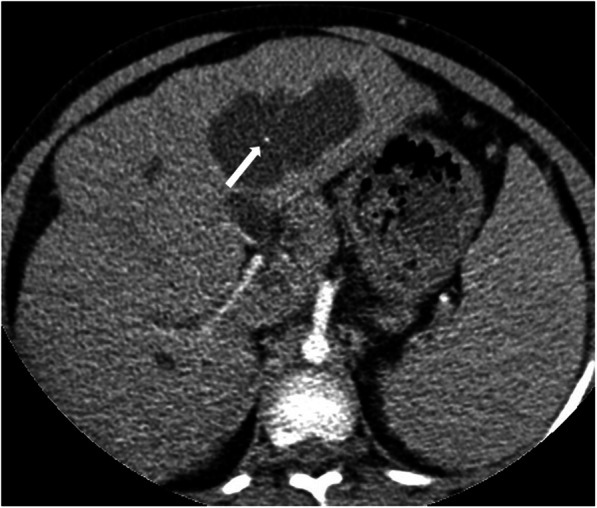
A 6-year-old male patient with known Caroli syndrome. Axial plane arterial phase CT image shows central dot sign (arrow)

**Fig 6:**
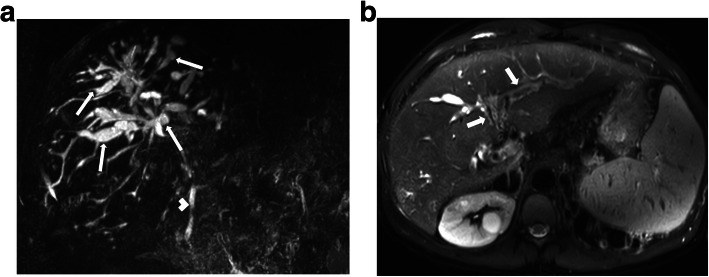
A 30-year-old male patient with known Caroli syndrome presenting with jaundice. **a** Coronal plane MRCP MIP (maximum intensity projection) image shows multifocal segmental dilation of the intrahepatic bile ducts (arrows). Choledoc is normal (arrowhead). **b** Axial plane T2W fat-saturated MR image shows increased T2 signal in the periportal area (arrows) and surface irregularity of the liver. These findings are consistent with congenital hepatic fibrosis

On imaging, cystic appearing enlarged intrahepatic bile ducts (in a saccular fashion) up to 5 cm in diameter is the classic finding. Endoluminal stones or sludge may be observed in these enlarged bile ducts. The detection of “central dot sign” is highly suggestive for CD (Fig. 5). This imaging finding refers to portal radicle within these dilated bile ducts and they tend to show strong enhancement after contrast injection [4]. Magnetic resonance cholangiopancreatography (MRCP) is helpful not only for detecting the presence of endoluminal abnormalities but also for demonstrating the extent of the disease (Fig. 6). The extrahepatic bile ducts are typically not affected and disease may be bilobar or limited to one lobe, mostly the left lobe. Surgical resection of the affected liver lobe or segment may be curative in limited disease. Hepatocyte-specific contrast agents may be helpful for demonstrating the communication between the parenchymal cysts and the biliary system.

### Choledochal cysts

Choledochal cysts are rare congenital malformations of the extrahepatic and intrahepatic biliary system. They are more common in females and the incidence is higher in Asian countries [5]. The most commonly accepted classification is proposed by Todani et al. in 1977 [6].

**Fig. 7:**
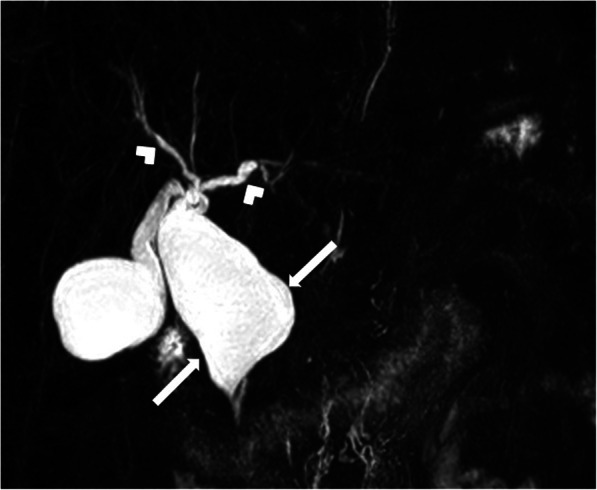
A 48-year-old female patient presenting with jaundice. Coronal plane MRCP MIP image shows fusiform dilation of the common bile duct (arrows). Also, note is made of mild dilation in the intrahepatic bile ducts (arrowheads)

Type I: This group has been subclassified into three subgroups with type IC being the most common. Type IA refers to diffuse cystic dilation of the extrahepatic bile duct whereas type IB is seen as focal cystic dilation in the extrahepatic bile duct. Type IC is characterized by diffuse fusiform dilation of the entire extrahepatic bile duct (Fig. 7).

**Fig. 8:**
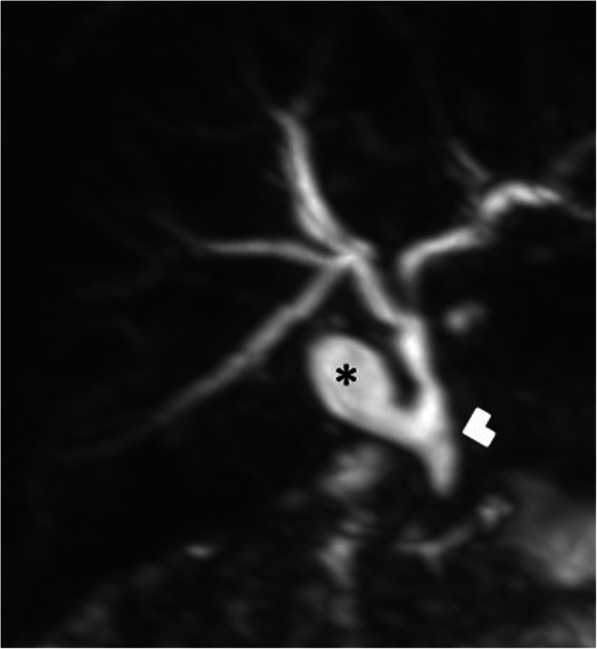
A 2-year-old female patient presenting with jaundice. On the US, a saccular outpouching arising from the supraduodenal extrahepatic bile duct was found (not shown). Coronal plane MRCP MIP image shows a diverticulum (asterisk), arising from the supraduodenal extrahepatic bile duct (arrowhead)

Type II: This type is the least common one with focal diverticular outpouching of the common bile duct (Fig. 8).

**Fig 9:**
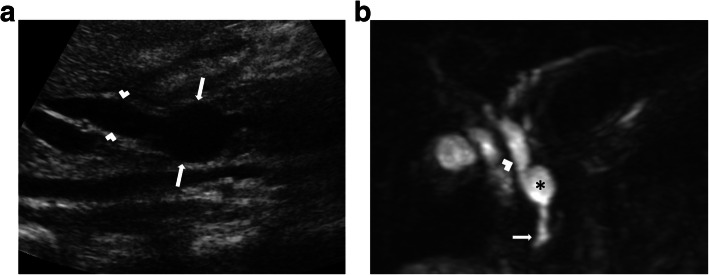
An 11-year-old female patient with newly diagnosed Hodgkin lymphoma underwent an initial US exam. **a** US image reveals choledochocele (long arrows) that involves the intramural segment of the distal common bile duct (arrowheads). **b** Coronal plane MRCP MIP image shows choledochocele (asterisk), common bile duct (arrowhead), and duodenum (arrow)

Type III: This group is also known as choledochoceles and refers to intramural dilation of the most distal portion of the common bile duct (Fig. 9).

**Fig. 10:**
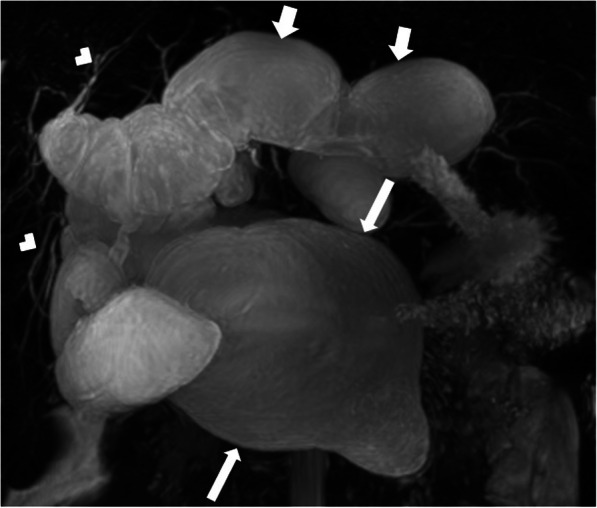
A 23-year-old pregnant patient presenting with jaundice. Coronal plane MRCP MIP image shows fusiform dilation of the entire extrahepatic bile duct (long arrows) with extensive dilation of the intrahepatic bile ducts in the left lobe (short arrows). Intrahepatic bile ducts in the right lobe are normal (arrowheads)

Type IV: This group has been subclassified into two subgroups. Type IVA is characterized by combined saccular-shaped dilations in the intrahepatic and extrahepatic bile ducts. Type IVB refers to saccular dilations restricted to extrahepatic bile ducts (Fig. 10).

Type V: This group is also known as Caroli disease.

Choledochal cysts typically manifest before the age of 10 years. The diagnosis is typically made after the clinical emergence of complications. Among these potential complications, cholangitis and pancreatitis are the most common. Malignancy may also be seen in the course of the disease. The risk of malignancy development is not rare (around 10–15% of the cases) with the extrahepatic biliary system and gallbladder being the most common [40, 41].

### Biliary hamartomas

Biliary hamartomas, also known as the “von Meyenburg complex (VMC)” was first described in 1918 and is characterized by the presence of multiple bile duct hamartomas [7]. Histopathologically, they are composed of abnormally dilated intrahepatic bile ducts embedded in a fibrous stroma. The prevalence of this abnormality is between 0.6% and 2.8% on autopsy studies [8]. This abnormality is asymptomatic in the majority of the patients and is incidentally detected [7]**.** The detection of biliary hamartomas may cause diagnostic confusion in patients having a history of cancer.

**Fig. 11:**
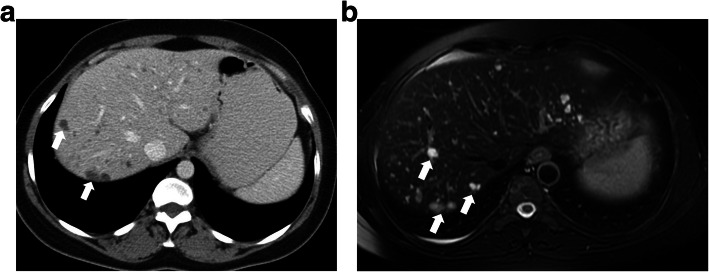
A 48-year-old female patient with known breast cancer underwent abdominal CT scanning for distant metastasis evaluation. **a** Axial plane post-contrast CT image shows multiple hypodense lesions (arrows) in both liver lobes. **b** Axial plane fat-saturated T2W MR image shows multiple cystic lesions (arrows) consistent with biliary hamartomas

These lesions appear as focal hypodense lesions on CT sometimes with irregular borders (Fig. 11). They typically do not enhance after contrast injection. T2-weighted (T2W) MR images are very helpful for diagnosis as biliary hamartomas are typically homogeneously hyperintense (Fig. 11) whereas they are seen as hypointense on T1W images with no apparent contrast enhancement on dynamic T1W 3D gradient echo sequences. The cysts are generally subcentimeter in diameter and innumerable spread throughout the liver parenchyma [7].

### Ciliated hepatic foregut cyst

Ciliated hepatic foregut cysts (CHFC) are generally asymptomatic and they are most commonly diagnosed incidentally on imaging or during surgery [42]**.** It has been proposed that CHFCs originate from intrahepatically entrapped detached hepatic diverticulum or abnormal tracheobronchiolar bud that may have migrated caudally at the early stages of the embryonic development of the foregut [43]. It is a very rare clinical finding and histopathologically, they are composed of ciliated, pseudostratified columnar epithelium, a layer of subepithelial connective tissue, a smooth muscle layer, and outer capsule [44]. Management strategy is controversial but a more aggressive approach such as surgical resection has been recommended as the malignant transformation has been reported in few cases [9]**.** They are more common in men and the medial segment of the left hepatic lobe (segments 4A and 4B) is the most common location.

**Fig. 12:**
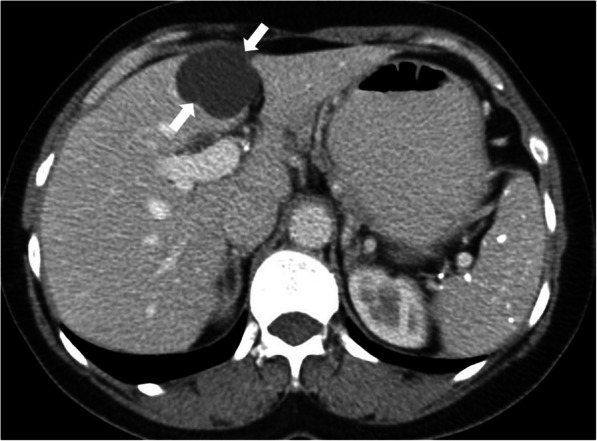
A 40-year-old female patient with known breast cancer. Axial plane post-contrast CT image shows a unilocular cystic lesion located in the subcapsular area of the segment 4 (arrows). The location was found to be typical for CHFC. The cyst was stable on 2-year follow-up study (not shown)

On US and cross-sectional imaging, they typically appear as unilocular cystic lesion located in the subcapsular area along the anterior surface of the liver (Fig. 12). The mean size is 3 cm, with a range of 1–12 cm [9]. They are seen as hypodense lesions on CT studies with a typical hyperintensity on T2W MR images. Enhancement is typically not detected after contrast injection. Characteristic location is an important diagnostic clue for diagnosis as signal characteristics are not different from usual benign hepatic cysts.

## Kidney

### Autosomal dominant polycystic kidney disease

Autosomal dominant polycystic kidney disease (ADPKD) is a relatively common disease and occurs in approximately 1/1000 individuals. This hereditary condition is largely inherited in autosomal dominant fashion. Most patients become hemodialysis dependent around the 5th to 7th decade of life [10].

**Fig. 13:**
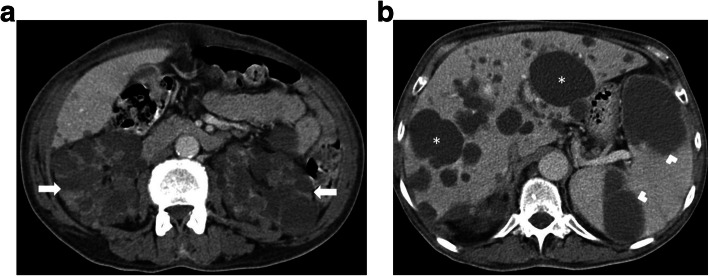
A 63-year-old male patient with known ADPKD. **a** Axial plane post-contrast CT image shows enlarged kidneys with innumerable cysts (arrows). **b** More cranial CT image demonstrates multiple hepatic (asterisks) and splenic cysts (arrowheads)

The cysts appear as typically hypoechoic on the US and hypodense on non-contrast CT images (Fig. 13). MRI findings are typical on advanced-stage disease with a bright T2 signal. Despite the fact that most of the cysts have typical imaging findings, some cysts may demonstrate unusual signal characteristics due to proteinaceous content. MRI studies with subtraction images may be helpful in the mural and cavitary evaluation of these cysts [10].

**Fig. 14:**
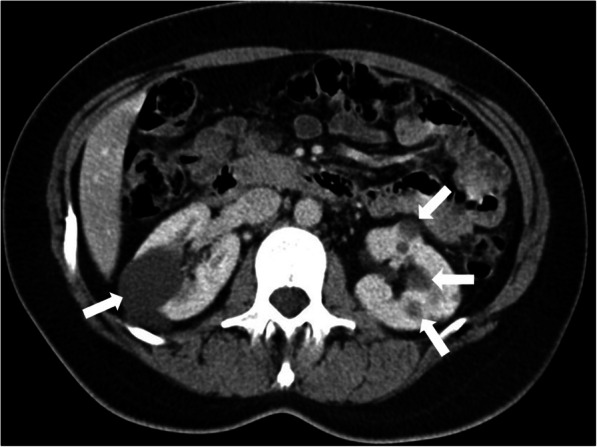
A 34-year-old female patient with known hypertension and family history of ADPKD. Axial plane post-contrast CT image shows bilateral renal cysts with different sizes (arrows). Normal renal parenchyma can be seen. Genetic analysis confirmed the ADPKD

**Fig. 15:**
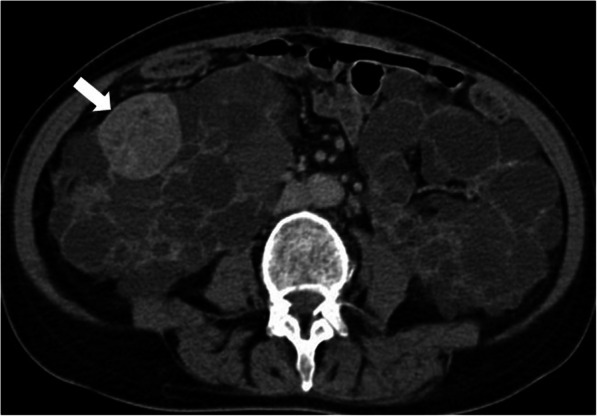
A 56-year-old female patient with known ADPKD. Axial plane postcontrast CT image shows enlarged kidneys with innumerable cysts and a solid mass in the right kidney (arrow). Histopathological examination after surgery revealed papillary type renal cell carcinoma

In the early stages of the disease, the kidneys may appear as either normal kidneys to single or multiple cysts in one or both kidneys (Fig. 14). The cysts typically increase in size and number into adulthood and during the final stages of the disease the entire renal parenchyma may be replaced with the cysts. Epidemiological and molecular biological data demonstrate that patients with ADPKD bear an increased risk for renal cell cancer (RCC). But preoperative image-based diagnosis is often challenging because of the distortion of the renal parenchyma [10]. (Fig. 15).

### Autosomal recessive polycystic kidney disease

Autosomal recessive polycystic kidney disease (ARPKD) is much less common than ADPKD and occurs in approximately one in 20.000 individuals [11]. The diagnosis is generally made in utero due to oligohydramnios from decreased fetal urine production. Severe renal dysfunction immediately after birth is typical. Abdominal distension due to enlarged kidneys, bilateral flank “masses”, and/or abdominal distension are common clinical findings. Pulmonary hypoplasia and pneumothorax are also common. Congenital hepatic fibrosis, Caroli disease, and bile duct ectasia may also be detected as associating abnormalities [12].

**Fig. 16:**
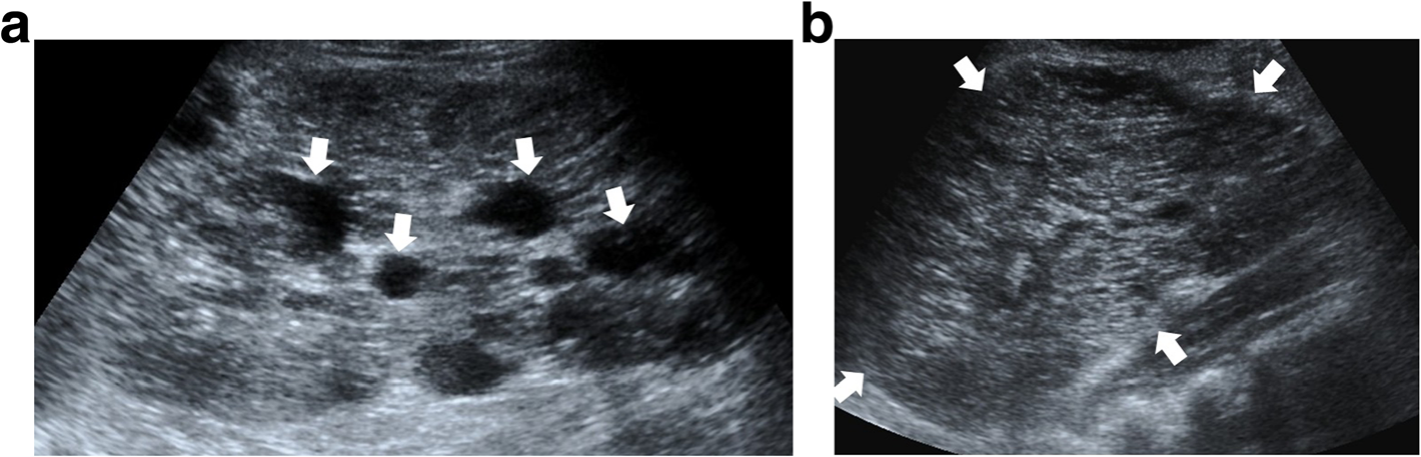
A 3-year-old male patient known ARPKD. **a** Sagittal view US image shows an enlarged kidney with cortical and medullary hyperechogenicity due to small cysts. There are also bigger cysts in the kidney (arrows). **b** Axial plane US image of the liver shows contour irregularities and parenchymal heterogeneity (arrows) consistent with congenital hepatic fibrosis

**Fig. 17:**
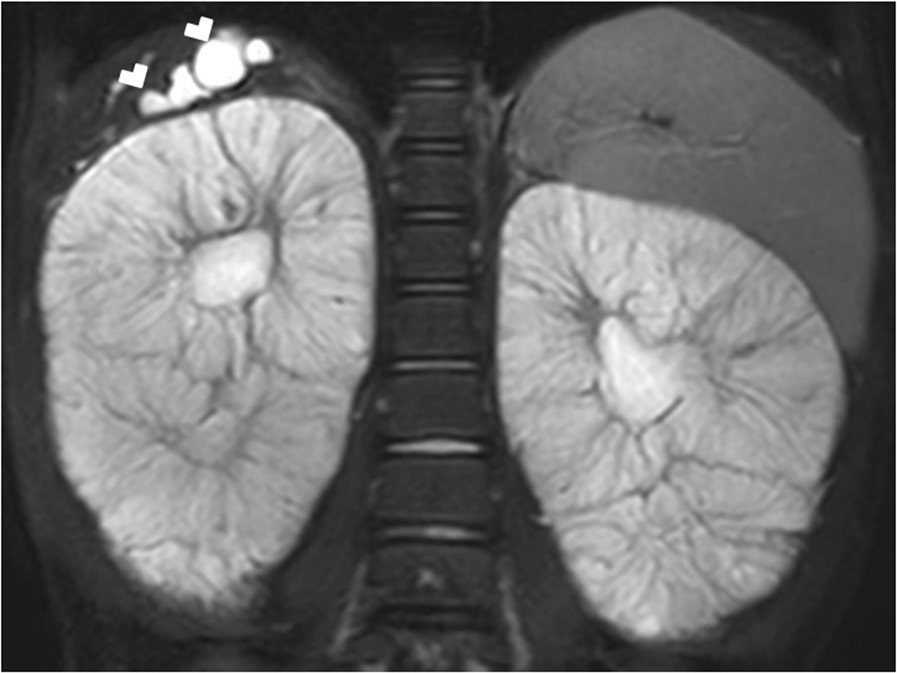
A 5-year-old male patient with known ARPKD. Coronal plane T2W MR image shows enlarged kidneys with diffusely increased T2 signal. Also note subcentimeter liver cysts (arrowheads)

The US is the most commonly used modality for diagnosis. Enlarged kidneys, with thickened parenchyma, is the typical finding. Renal medulla appears as hyperechoic due to the presence of several ectatic renal tubules, also called as microcysts [11]**.** Larger cysts (larger than 1 cm) may be seen in a certain subset of patients [13] (Fig. 16)**.** With the advancement of the age, the cysts typically enlarge and may replace the renal parenchyma. On US studies, findings suggestive of hepatic abnormalities should also be sought after for early diagnosis and intervention. CT and MRI are rarely needed for diagnosis and IV contrast is not typically used due to limited renal reserve. Non-complicated cysts appear as hypodense on CT and T2 hyperintense on MR examinations (Fig. 17).

### Multicystic dysplastic kidney disease

Multicystic dysplastic kidney (MCDK) is a congenital non-heritable cystic disease of the kidneys in the pediatric patient group. Renal cysts are formed in utero and may be observed on antenatal US examinations. The disease is typically unilateral and the affected kidney typically does not function [45].

**Fig. 18:**
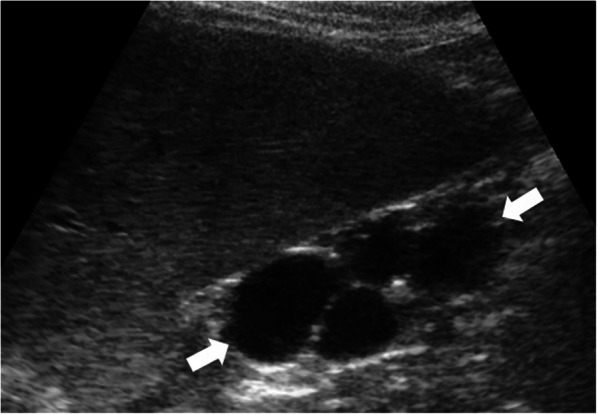
Newborn with prenatally detected left kidney cysts underwent an US study on the first day of life. Sagittal view US image shows multiple cysts in the left renal fossa with no discernible normal renal parenchyma. The right kidney was normal (not shown). Imaging findings were found to be consistent with MCDK

**Fig. 19:**
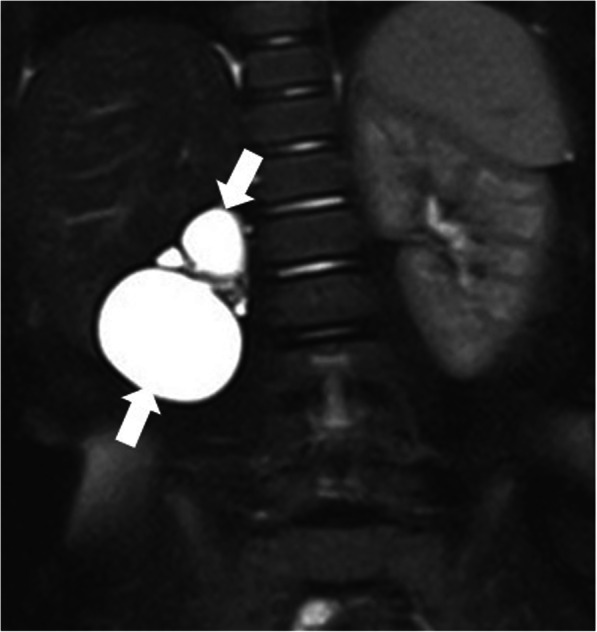
A 12-day-old newborn with prenatally diagnosed right kidney cysts underwent an abdominal MRI examination. Coronal plane T2W MR image shows multiple cysts completely replacing the right kidney parenchyma (arrows). There was no discernible renal parenchyma aside from the cysts. The left kidney was normal

The US is typically the imaging modality of choice in this patient group. The detection of renal cysts in a disorganized pattern is the typical imaging finding and renal parenchyma is characteristically completely replaced by these cysts [14] (Fig. 18). MRI demonstrates similar findings with the US exam (Fig. 19).

### Nephronophthisis and medullary cystic kidney disease

Medullary cystic kidney disease and nephronophthisis, which are a common cause of end-stage renal disease during the first 3 decades of life, are inherited diseases with similar renal morphology and histopathologic features. The inheritance pattern is variable; nephronophthisis is autosomal recessive in inheritance and medullary cystic kidney disease is autosomal dominant [15]. Several other syndromes may be associated with nephronophthisis [46].

**Fig. 20:**
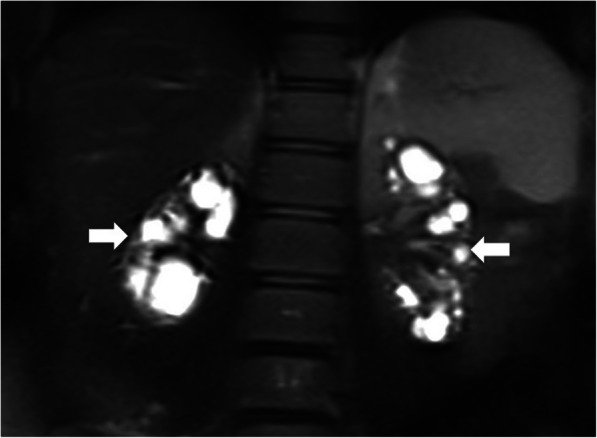
A 13-year-old female patient with known histopathologically proven nephronophthisis now presenting with flank pain. The patient is on chronic hemodialysis. Coronal plane T2W MR image shows multiple cysts in both kidneys located at the corticomedullary junction. There was no discernible healthy renal parenchyma in the kidneys. Also note that both kidneys (arrows) are small in their overall sizes in contrast to ARPKD

On US studies, the kidneys, contrary to ARPKD, appear small due to parenchymal fibrosis. Cysts, of varying size, in medullary and corticomedullary locations may be detected [15, 16] (Fig. 20). Early in life, it may be possible not to detect any cysts, the kidneys may appear hyperechoic with the loss of corticomedullary differentiation [47]. The cystic changes in the renal parenchyma are generally progressive and advance with age.

### Von Hippel–Lindau disease

Von Hippel–Lindau (VHL) syndrome is a phakomatosis inherited in an autosomal dominant fashion. It is a rare disease which affects 17/36.000–53.000 individuals [48]. This syndrome predisposes the affected individual to different cancers.

Renal manifestations are common and renal cysts may be detected in 59–63% of patients and bilateral involvement is extremely common (around 75% of the cases) [17]. RCCs are also common and are seen in 24–45% of the affected individuals [49]. Periodic screening of the kidneys is mandatory as untreated RCCs carry a poor prognosis with a tendency to metastasize [18]. The histopathologic features of the renal cysts vary, ranging from simple and hyperplastic cysts to cystic clear cell carcinomas and to solid tumors [50]. All mentioned benign and malignant lesions may occur in the same kidney at the same time. Tumors may arise from precursor cystic lesions or completely de novo [49]. Thus, continuous imaging screening is fundamental for early diagnosis and treatment to prevent metastatic disease. Cysts may enlarge or regress with time leaving parenchymal scars, and no relationship has been observed with the cyst size and number and the malignant potential [17].

**Fig. 21:**
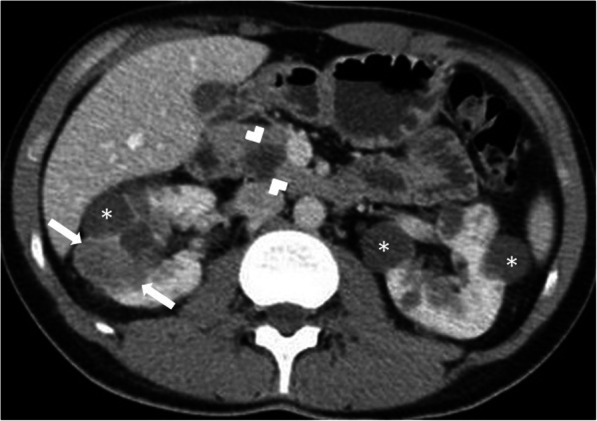
A 43-year-old male patient with known VHL underwent a follow-up CT exam. Axial plane post-contrast CT image shows several renal cysts (asterisks) in both kidneys. Also note is made of a new focus of clear cell RCC (surgically confirmed) in the right kidney (arrow). A pancreatic cyst in the uncinate process was also detected (arrowheads)

The US may be helpful to differentiate the cysts from the solid masses but CT and MRI are more commonly used for both diagnosis and follow-up (Fig. 21). Mural nodules within the cyst walls are suggestive for cancer and they can be observed with relative ease on both CT and MRI. Pure solid lesions may also be assessed with these two modalities and renal vein involvement can also be detected. CT and MRI are more sensitive for detecting small lesions (2 cm) [49]**.** MRI might be preferable for follow-up purposes, especially in young patients, due to lack of radiation with this modality. Subtraction images may be helpful for differentiating proteinaceous cysts from solid masses. Due to the progressive course of VHL syndrome, partial nephrectomy and percutaneous ablative measures are common approaches for the treatment of RCCs in these patients.

### Tuberous sclerosis complex

Tuberous sclerosis complex (TSC) disease affects around 1/5.000–10.000 individuals [51]**.** TSC is typically inherited in an autosomal dominant fashion but the expression is variable. Around 66% of the cases are secondary to sporadic mutation**.** TSC is a multisystemic disease and may manifest with numerous mesodermal and ectodermal abnormalities. Kidneys are also affected along the course of the disease. Angiomyolipomas, with variable fat content, and renal cysts are the two most common renal lesions in these patients [19]. Large angiomyolipomas may spontaneously rupture in the course of the disease, renal cysts are almost always asymptomatic [52]**.** Angiomyolipomas tend to be more numerous and common compared to renal cysts [19].

**Fig. 22:**
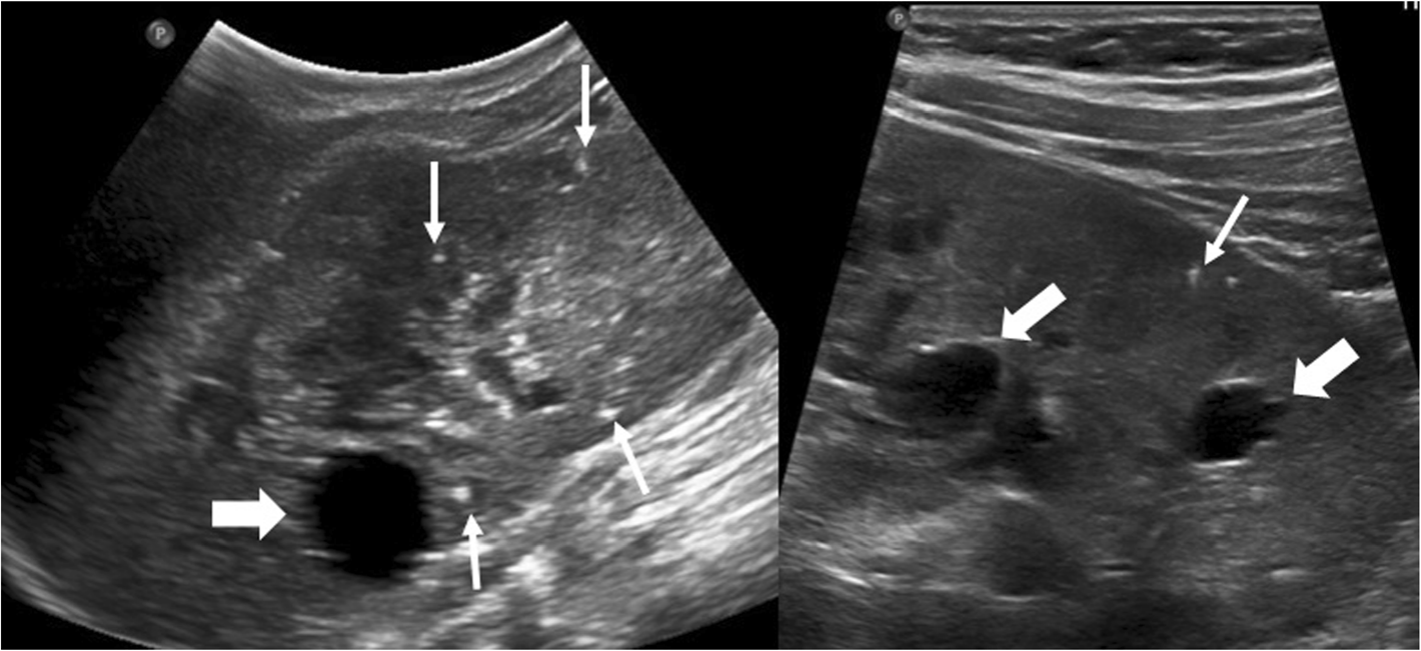
A 9-year-old male patient with known TSC underwent follow-up US exam. US images show punctate hyperechoic foci consistent with subcentimeter angiomyolipomas (fine arrows). Also note is made of several parenchymal cysts (thick arrows)

On imaging, renal cysts in TSC appear like simple renal cysts (Fig. 22). Differential diagnosis from ADPKD may be difficult as fewer cysts are typically seen at the early stage of this disease. The very common association of renal cysts with angiomyolipomas may serve as a reliable imaging clue for correct diagnosis. The cysts tend not to be large in size and the average diameter of these cysts was reported to be around 20 mm which may be another helpful clue considering the large sizes of the cysts in patients with ADPKD [19].

## Pancreas

### Von Hippel-Lindau disease

As VHL is a multisystemic disease, the pancreas may also be affected among the course of the disease. The pancreatic manifestations are either cysts, serous cystadenomas, or neuroendocrine tumors (NETs). Combined lesion pattern, the presentation of solid and cystic manifestations, may be observed in 11.5% of patients with VHL disease and in 7.6% of the cases pancreas may be the only affected organ [20].

**Fig. 23:**
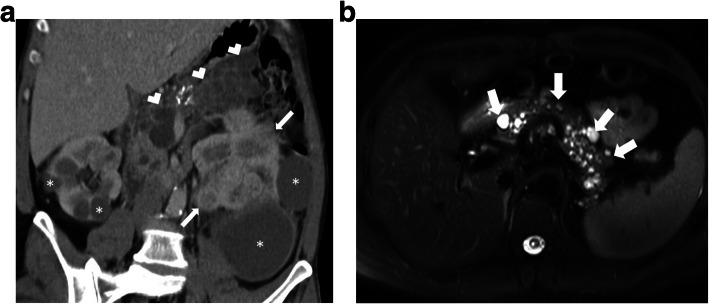
Two different patients with VHL. **a** Coronal plane post-contrast CT image of a 47-year-old male patient shows innumerable pancreatic cysts (arrowheads). Also note is made of large-sized solid RCC (arrows) in the left kidney and bilateral renal cysts (asterisks). **b** Axial plane T2W MR image of a 40-year-old male patient demonstrates multiple cysts of the pancreas, almost completely replacing pancreatic parenchyma (arrows)

**Fig. 24:**
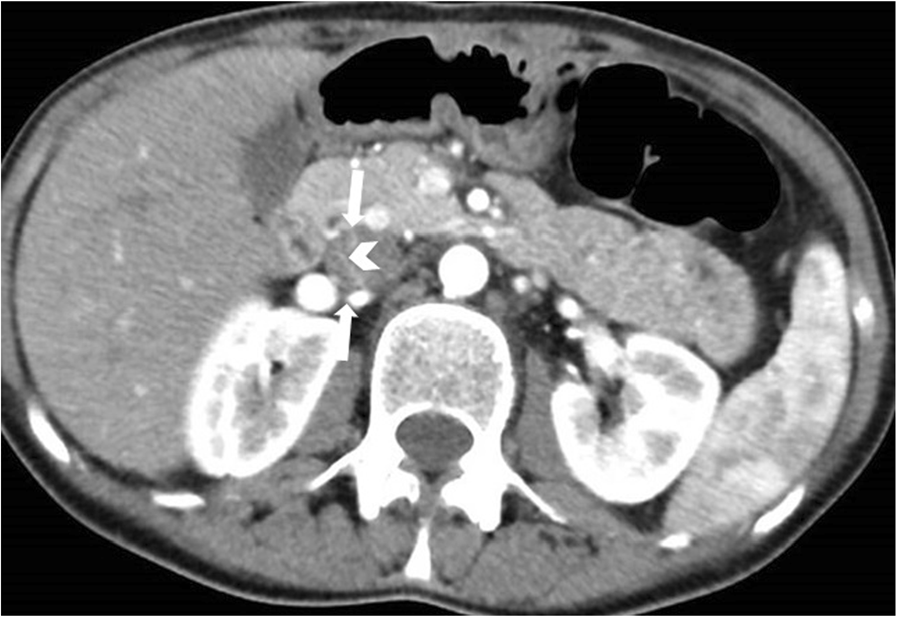
A 21-year-old female patient with recently diagnosed (with genetic testing after a recent diagnosis of her elder sister) VHL underwent a baseline CT exam. Axial plane post-contrast pancreatic phase CT image shows a predominantly cystic lesion in the uncinate process (arrows) with enhancing mildly thickened internal septation (arrowhead). The lesion was found to be highly suspicious of a cystic NET. Surgical removal and histopathological examination confirmed the presence of cystic NET

The pancreatic cysts follow the typical imaging characteristics of cysts elsewhere in the body (Fig. 23). Pancreatic NETs are mostly solid but cystic tumors were also reported [21] (Fig. 24)**.** These tumors, be it cystic or solid, generally tend to have early arterial phase-contrast enhancement for detection and correct characterization.

### Multiple endocrine neoplasia type I

MEN 1 is an inherited endocrine tumor syndrome in autosomal dominant fashion. The pituitary gland, islet cells of the pancreas, and parathyroid glands are the most common tumor sites. Imaging plays an important role in the diagnosis and management of the disease [53].

**Fig. 25:**
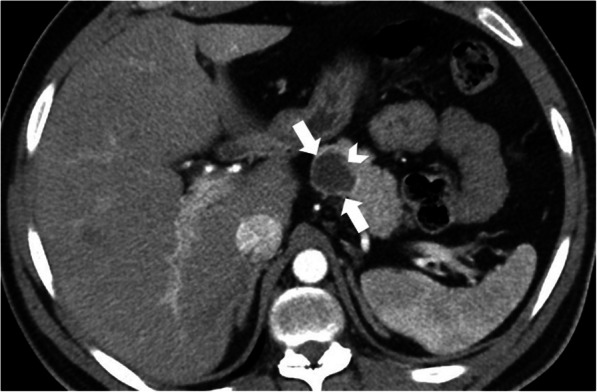
A 31-year-old male patient with known MEN-1 syndrome underwent CT scanning to detect manifestations of the syndrome. Axial plane post-contrast pancreatic phase CT image shows a predominantly cystic lesion in the pancreatic body (arrows). Focal contrast-enhancing mural wall thickening can also be seen (arrowhead). Histopathological findings revealed cystic NET

Most pancreatic NETs in MEN 1 are functional with the gastrinoma being the most common [54]. The pancreatic NETs seen in MEN 1 follow the typical imaging characteristics of spontaneous NETs. Rarely, the tumors can be cystic [22] (Fig. 25).

### Cystic fibrosis

Cystic fibrosis (CF) is a common hereditary systemic disease. Exocrine glands are commonly affected and pancreatic insufficiency is a common manifestation of the disease.

Four different imaging patterns were described in patients with CF: (1) partial fatty replacement of the pancreas, (2) complete fatty replacement of the pancreas, (3) atrophy of the pancreas, and (4) pancreatic cystosis. Among these four different patterns, pancreatic cystosis is the least common [55]**.** In pancreatic cystosis, the organ parenchyma is filled with macrocysts and the development of these cysts has been linked to bicarbonate transport [23].

**Fig. 26:**
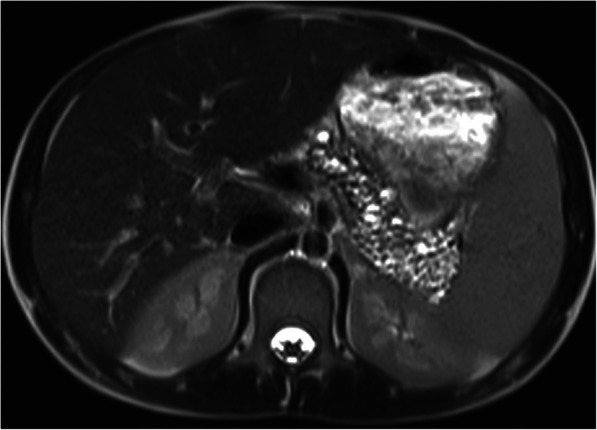
A 13-year-old male patient with known CF and pancreatic insufficiency. Axial plane T2W MR image reveals innumerable cysts of subcentimeter size scattered throughout the pancreas, almost completely replacing the parenchyma (arrows)

On the US, the cysts appear as hypoechoic structures with no associating mural wall thickening and nodularity. The size of the cysts is variable ranging from 0.5 cm to 1.2 cm in diameter. Vascular displacement due to the mass effect of these cysts are may be seen [56]. MRI is also a very helpful modality to detect the cysts and their anatomic relationship, due to its high soft-tissue resolution (Fig. 26). Malignant transformation of these cysts has not reported before [57]. Polycystic kidney disease and VHL may be considered in differential diagnosis but detection of normal kidneys and patient history are generally diagnostic without any significant confusion.

## Gastrointestinal tract duplication cysts

Gastrointestinal (GI) tract duplication cysts are rare congenital malformations which are typically detected in young patients and adults [58]. They may occur anywhere along the GI tract but distal ileum is the most commonly affected segment followed by the esophagus, colon, jejunum, stomach, and the duodenum [59]. These lesions may be contained within the wall of the affected segments but may also be detected extrinsic to the bowel segment and may appear as either spherical (80%) or tubular cysts (20%) [60, 61]. This morphologic difference may provide a clue regarding the possible communication between the enteric lumen and the duplication cysts, as the spheric ones generally do not communicate with the lumen, whereas, its tubular counterparts typically do.

Histopathologically, GI tract duplication cysts consist of an epithelial lining containing the mucosa of the GI tract and a surrounding smooth muscle. The cyst is also closely attached to the enteric wall [24].

GI tract duplication cysts are most commonly diagnosed incidentally but complications may also occur, including obstruction, volvulus, intussusception, bleeding, perforation, and infection. Malignant transformation from the mucosa is extremely rare and surgical resection is the preferred approach for treatment [59].

**Fig. 27:**
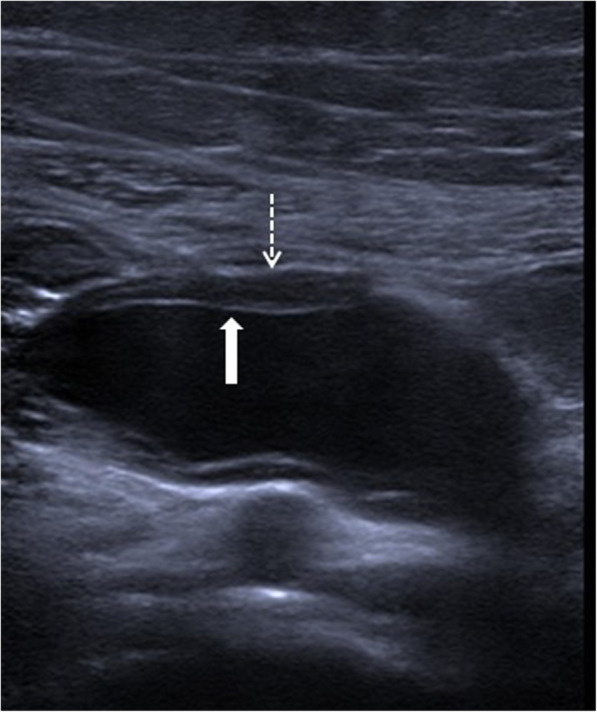
An 8-year-old male patient presenting to the emergency department (ED) with right lower quadrant pain. Axial view US image shows a cystic mass with thick wall suggested for “double-wall” sign: The mucosa appears hyperechoic (arrow) whereas the muscular layer hypoechoic (dashed-line arrow). Laparoscopic surgery and histopathologic examination confirmed the ileal duplication cyst causing intestinal obstruction

**Fig. 28:**
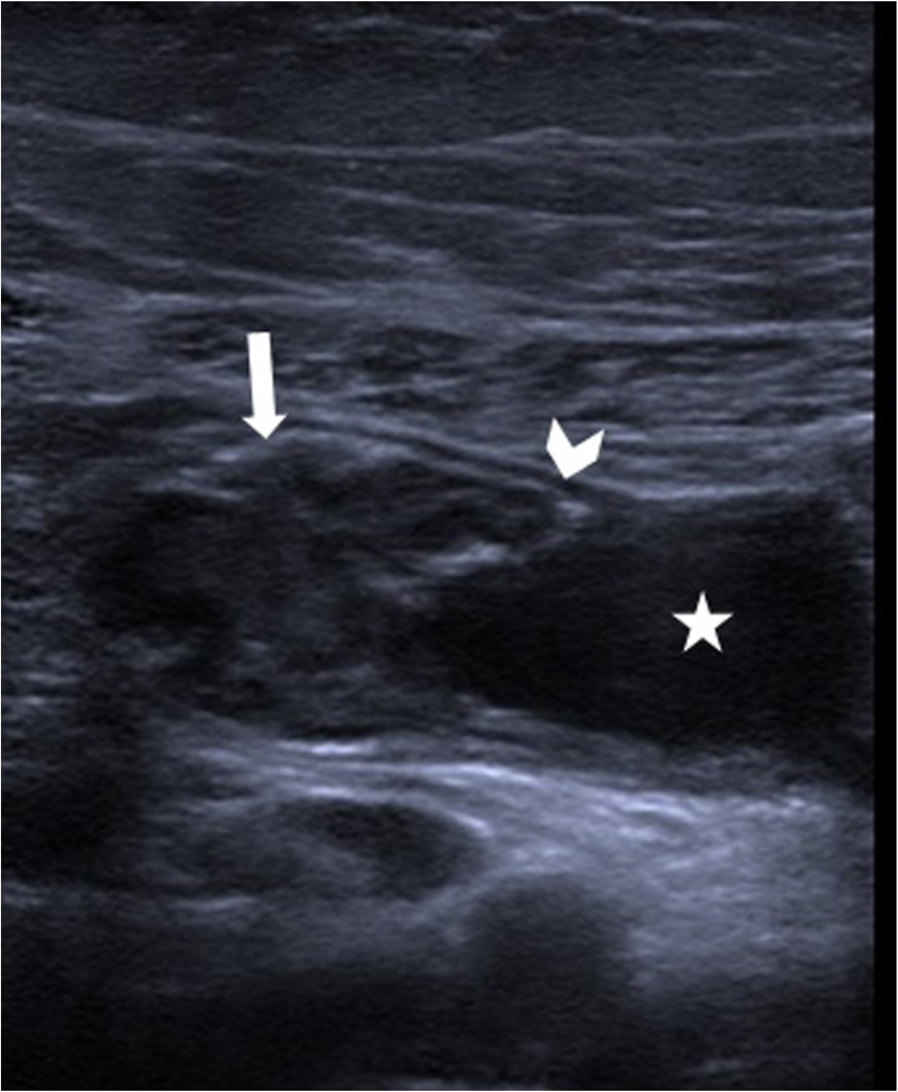
A 7-year-old female patient presenting to ED with right lower quadrant pain. Axial view US image demonstrates a purely cystic mass (star) in the right lower quadrant. The wall of this lesion was in direct continuity (arrowhead) with the adjacent terminal ileum segment (arrow)

The US is the most commonly used imaging modality. The diagnosis is generally straightforward when the cyst within the close proximity of the bowel segment. The double-wall or muscular rim sign is the typical imaging finding (Fig. 27). This imaging appearance is due to the inner hyperechoic mucosa and the surrounding hypoechoic smooth muscle layer (muscularis propria) [24]. The GI tract duplication cysts share a common wall with the adjacent gut segment. The so-called “Y configuration” is a helpful diagnostic sign that is indicative of a shared wall with the cyst and the neighboring bowel wall (Fig. 28). This sign is caused by the splitting of the shared muscularis propria with the cyst and bowel wall [25, 26]. Due to the presence of smooth muscle content, these cysts may change shape due to muscular contractions of the cyst wall, which is another useful finding for diagnosis on real-time sonographic examination [58]. The cysts are generally homogenous on the sonographic exam; however, internal septations or luminal debris may also be observed in certain patients. Color Doppler US exam may be helpful to detect wall inflammation in complicated duplication cysts [24].

**Fig. 29:**
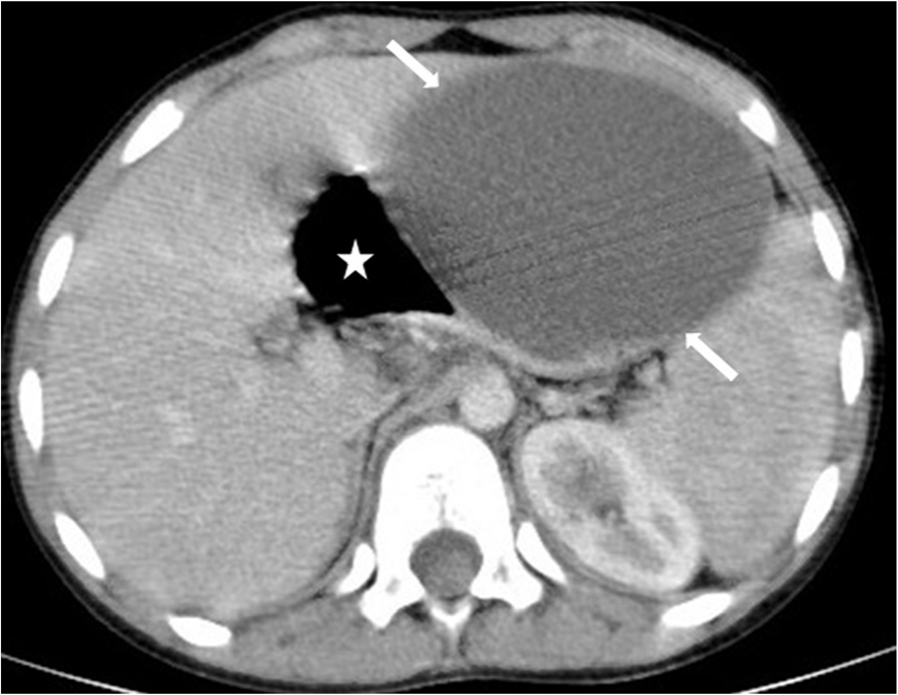
A 9-year-old female patient with palpable epigastric mass was found to have a large cystic mass in the epigastrium on US examination (not shown). An abdominal CT study was planned for better assessment of the anatomic relationship of this lesion. Axial plane post-contrast CT image shows a huge cystic mass (arrows) compressing and displacing the stomach (star). Surgery confirmed gastric duplication cyst

CT and MRI are not typically used for diagnosis due to ionizing radiation and increased patient cooperation, consecutively. However, these modalities may be used for better assessing the anatomical relations of duplication cysts (Fig. 29). They can also be used in patients with questionable malignant degeneration within these cysts.

## Abdominal lymphatic malformations

Abdominal lymphatic malformations (LMs) are rare congenital malformations of the lymphatic system. They are most commonly located in the small bowel mesentery, followed by omentum, mesocolon, and retroperitoneum [62]. Abdominal LMs are rare as LMs are most commonly located in the head-neck region [63]. LMs are generally small in size and most commonly diagnosed incidentally on imaging but symptoms may occur, including abdominal pain or distension, particularly in large size LMs. Asymptomatic LMs do not require treatment and can be followed with serial imaging. The common approach for the treatment of symptomatic LMs is surgical resection [27, 64, 65].

**Fig. 30:**
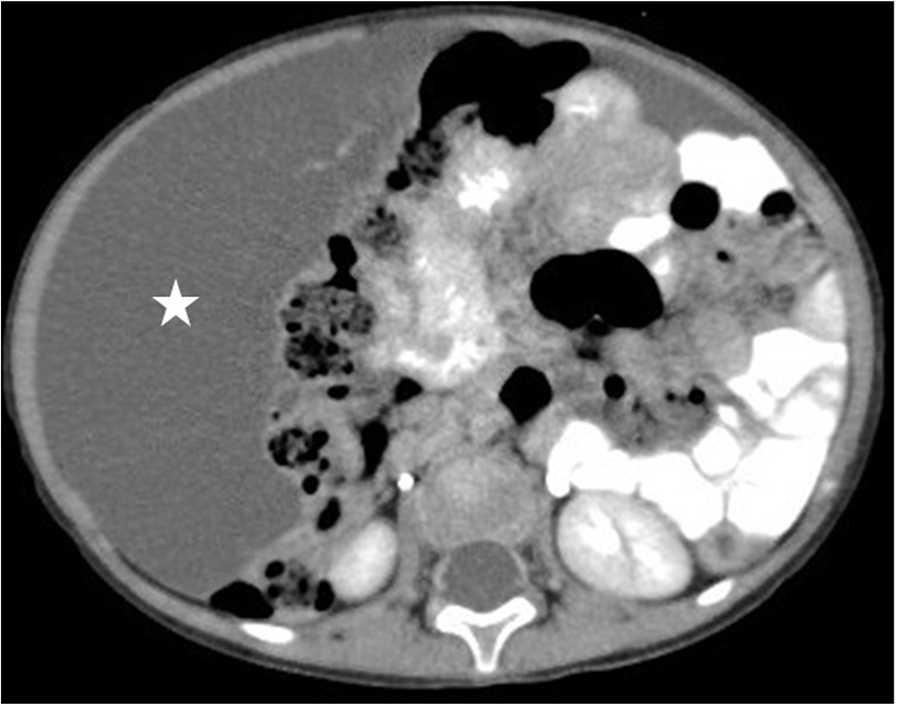
A 1-year-old male patient presented with progressively enlarging abdominal girth**.** Axial plane CT image after IV and oral contrast use shows a large purely cystic mass (star), displacing the bowel loops to the left side of the abdomen. Histopathological examination after surgical extirpation confirmed omental lymphangioma

**Fig. 31:**
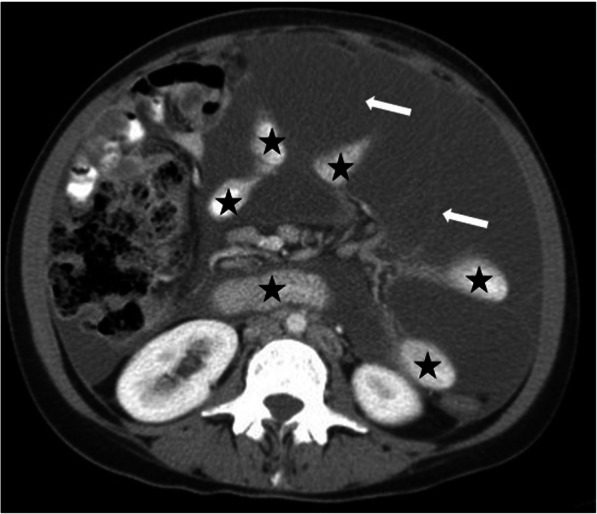
A 39-year-old female patient with progressive abdominal distension underwent an US exam which showed large volume ascites in the abdomen. The clinical and imaging findings were concerning for peritoneal carcinomatosis. Axial plane CT image after IV and oral contrast use reveals a large cystic lesion insinuating between the mesenteric leaves. This cystic mass was separating apart the adjacent bowel loops (stars). Also noted were internal septations (arrows) within this large cystic mass. Surgical and subsequent histopathological examinations confirmed large mesenteric lymphangioma.

**Fig. 32:**
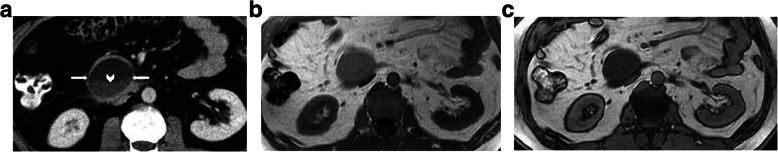
A 41-year-old female patient with newly diagnosed breast cancer underwent a staging body CT examination. **a** Axial plane post-contrast CT image shows a well-circumscribed retroperitoneal mass (arrows) with a fat-fluid level (arrowhead). **b, c** Abdominal MRI exam of the same patient showed signal loss within the cyst content on out-of-phase image (**c**) compared to in-phase image (**b**). Imaging findings were found to be compatible with chylous fluid containing lymphangioma. Follow-up imaging studies confirmed the stability of this lesion

US and CT are the two most commonly used modalities for diagnosis. The anatomic relations of these cystic masses and their internal contents may be assessed with high precision with both techniques (Fig. 30). Ascites may be confused with these LMs but well-circumscribed morphology, as opposed to free-floating ascites, favors LMs over ascites. However, LMs may also conform to omental anatomy and differential diagnosis from loculated ascites may be difficult in certain cases (Fig. 31). The fluid in the LMs may contain fat which may be better appreciated on CT or MRI studies [28] (Fig. 32). Small LMs are frequently mobile and, therefore, they can be observed in different locations on separate follow-up imaging studies [27].

## Diaphragm

### Diaphragmatic mesothelial cyst

Diaphragmatic mesothelial cysts (DMC) are derived from coelomic remnants and are lined with mesothelial cells [66]. Mesothelial cysts may be found in several places including the diaphragm. Due to its close proximity of DMCs to liver, lung, and pleura, it may be difficult to determine the diaphragm as the source organ.

**Fig. 33:**
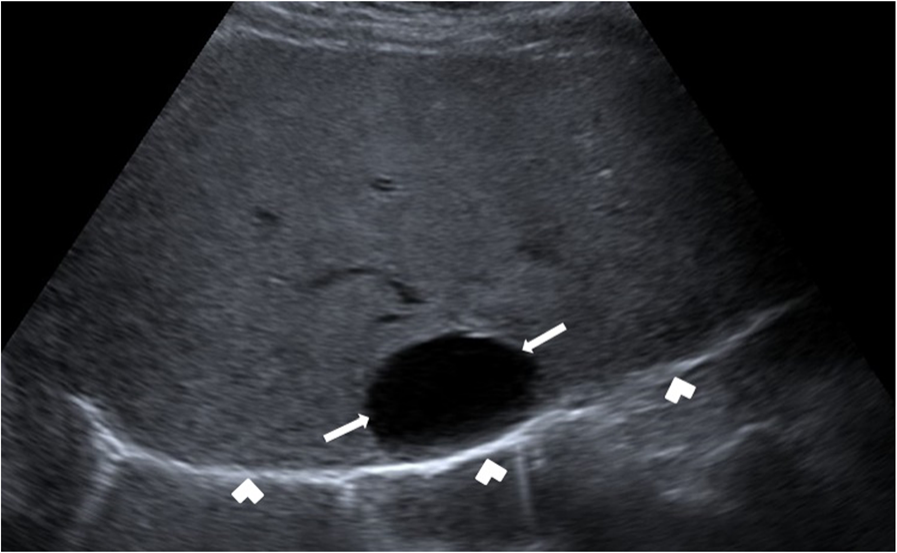
A 23-year-old female patient with recently diagnosed hepatitis B infection underwent an initial liver US exam. Axial view US image shows a oval-shaped purely cystic lesion (arrows) in the posterolateral aspect of the right liver lobe in close proximity to the right hemidiaphragm (arrowheads). The lesion was found to be representing a diaphragmatic mesothelial cyst. Follow-up studies confirmed the stability of this lesion

On imaging, they have characteristic findings of an ordinary cyst located elsewhere in the body. The walls of these cysts are thin with no associating solid component. They may also appear as bilobulated on sonography [29]**.** On the US, they appear as homogenously hypoechoic lesions located between the posterolateral aspect of the right liver lobe and the adjacent diaphragm (Fig. 33). CT and MRI may also be used as confirmatory studies. In these studies, internal content of the lesion may be better appreciated. Bronchogenic cysts, hydatid cysts, or an ordinary liver cyst may be considered in differential diagnosis. Bilobulated morphology of the cyst is an important clue for the diaphragmatic origin of the cyst [67]. These cysts may be effectively treated with a percutaneous approach [29].

## Prostate

### Prostatic utricle cyst—Mullerian duct cyst

Unlike paramedian ejaculatory ductus cysts, prostatic utricle and Mullerian duct cysts are two different cystic entities which are both located midline [68]. Prostatic utricle cysts may be associated with several genitourinary abnormalities [30]. On the contrary, Mullerian duct cysts are not expected to associate with any congenital genitourinary malformations. Both cysts may manifest with various symptoms, including difficulty urinating, dysuria, ejaculatory impairment, and hematospermia [31, 69]. In these cystic lesions, the mechanism of hematospermia is considered to be due to ejaculatory duct obstruction [68].

**Fig. 34:**
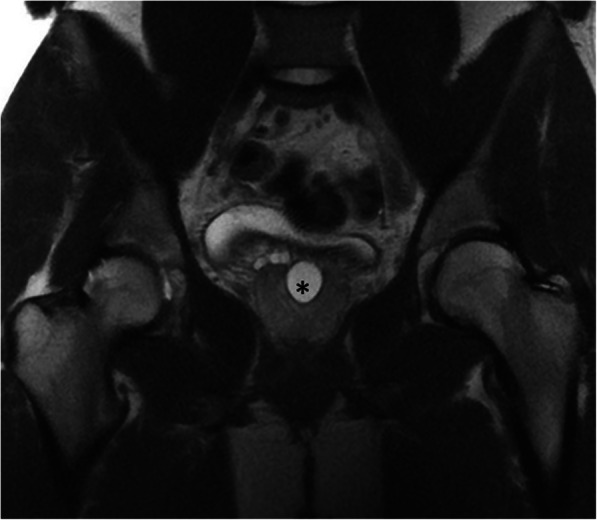
A 17-year-old male patient with urinary incontinence. Coronal plane T2W MR image shows a midline prostatic cyst (asterisk) that does not extend above the prostate gland

**Fig. 35:**
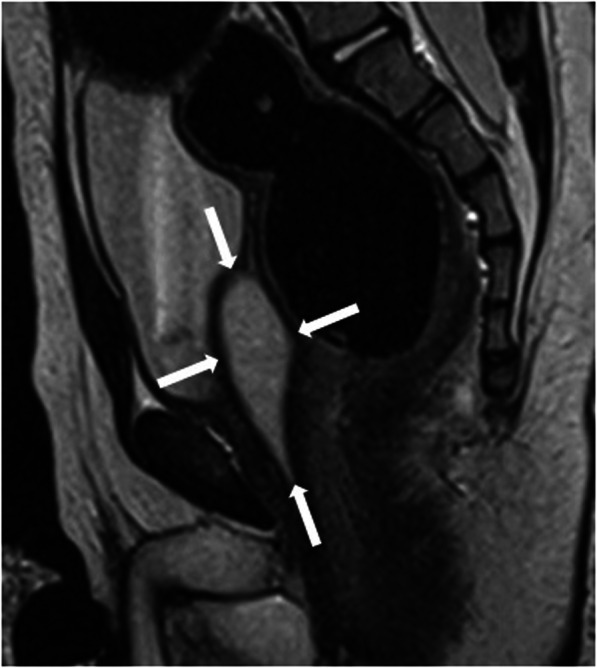
A 2-year-old male patient with recurrent urinary tract infection underwent a US exam and a midline cystic lesion was seen posterior of the bladder (not shown). Confirmatory MRI exam shows a tear-drop shaped midline cystic lesion (arrows) on the sagittal plane T2W image. The cystic lesion was extending above the posterior superior margin of the prostate, highly suggestive for the Mullerian duct cyst

**Fig. 36:**
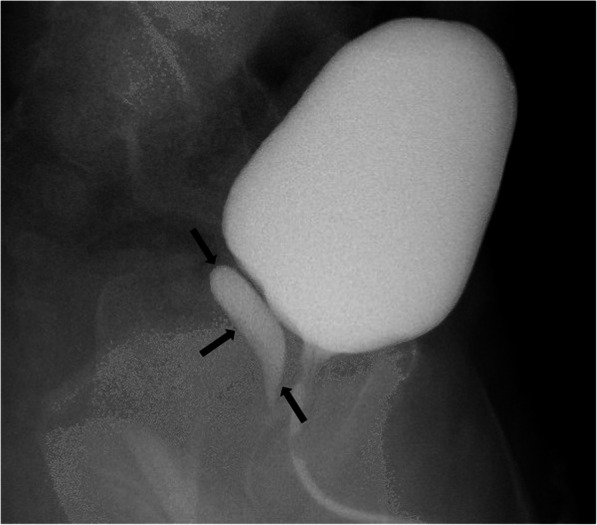
A 5-year-old male patient with recurrent urinary tract infection. Voiding cystourethrogram demonstrates a contrast filling prostatic utricle cyst (arrows)

Differentiation of these entities by imaging alone may be difficult. Typically, prostatic utricle cysts do not extend above the base of the prostate (Fig. 34), whereas Mullerian duct cysts are characteristically observed as teardrop-shaped midline cysts extending above the superior margin of the prostate (Fig. 35). In terms of size, prostatic utricle cysts are typically smaller than Mullerian duct cysts. From an anatomical standpoint, prostatic utricle cysts communicate with the prostatic urethra unlike Mullerian duct cyst [31, 68, 69]**.** Voiding cystourethrography may help differential diagnosis by demonstrating the connection between the prostatic utricle cyst and the urethra [70] (Fig. 36).

## Urachal cyst

The urachus is a ductal remnant that originates from the involution of the allantois and cloaca. It extends from the bladder dome to the umbilicus. In the late stages of gestation, it involutes and obliterates and observed as a median umbilical ligament in the postgestational period. The failure of this mentioned involution may result in persistent of this canal after birth. The most common types of this failed involution are patent urachus and urachal cyst [71].

Urachal cysts form when both the umbilical and bladder ends of the urachus are obliterated with nonobliterated segment in between. These urachal cysts are typically small and asymptomatic; however, infection of the cysts may cause symptoms [32, 71].

**Fig. 37:**
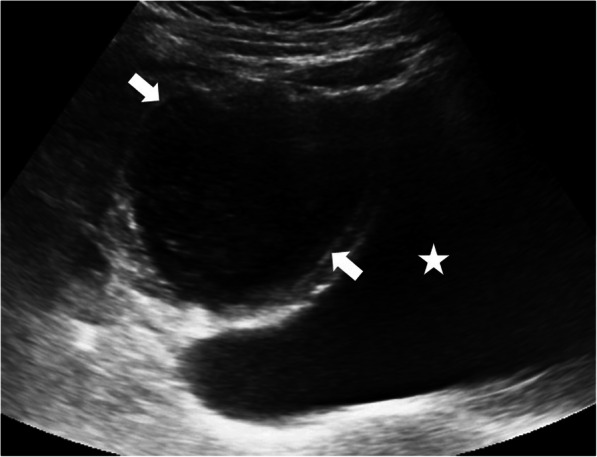
A 6-year-old female patient presenting to ED with suprapubic pain. Sagittal view US image shows a round-shaped cystic lesion (arrows) above the superior surface of the bladder (star). Due to the mixed echogenicity of the lesion, it was considered an infected urachal cyst. Surgical findings confirmed the diagnosis

On imaging, the diagnosis of an uncomplicated cyst may be easily made by detecting the cyst in the midline along the trajectory of the urachus. The detection of inhomogeneous cyst content and inflammatory stranding adjacent to the cyst may indicate infection [32] (Fig. 37).

## Zinner’s syndrome

Zinner’s syndrome is a rare developmental anomaly. It refers to the triad of ipsilateral ejaculatory duct obstruction, seminal vesicle cysts, and renal agenesis. The patients usually admit to the hospital with genitourinary symptoms in the 2nd–3rd decades of their life [72].

**Fig. 38:**
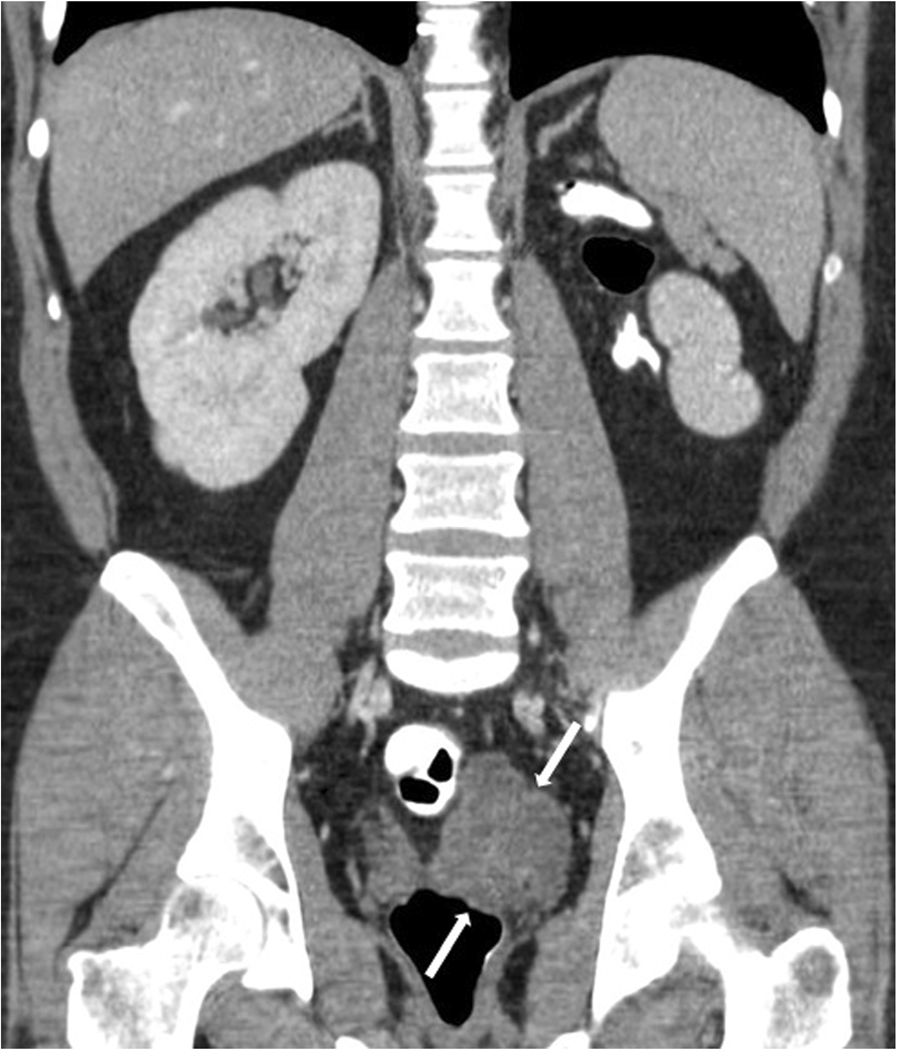
A 31-year-old male patient presenting with left groin pain. Coronal plane postcontrast CT image demonstrates left renal agenesis and left seminal vesicle cysts (arrows)

On imaging, the obstructed ejaculatory ducts are seen as tubular structures in the pelvis and the ipsilateral kidney is typically agenetic (Fig. 38). The content of ejaculatory ducts is characteristically anechoic on the US and homogenously hyperintense on T2W MR images. In the case of hemorrhage or infection, the duct content may appear as bright on T1W MR images. The detection of the tail-like connection between the cystic tubules and the seminal vesicle may indicate that the seminal vesicle is the site of origin [33].

## Conclusion

Congenital and hereditary cystic lesions of the abdomen are relatively rare. They may be diagnosed incidentally or may give rise to symptoms which prompt their diagnosis. Correct diagnosis is critical as they may simulate several other benign and malignant acquired diseases of the abdomen, all of which have very different treatment approaches and prognostic implications. With the correct and appropriate use of imaging, with relevant clinical information and patient history, diagnosis may be relatively straightforward and clinical management may be implemented appropriately.

## Data Availability

Data sharing is not applicable to this article as no datasets were generated or analyzed during the current study.

## Abbreviations

ADPKD: Autosomal dominant polycystic kidney disease
ARPKD: Autosomal recessive polycystic kidney disease
CD: Caroli disease
CF: Cystic fibrosis
CHFC: Ciliated hepatic foregut cyst
CT: Computed tomography
DMC: Diaphragmatic mesothelial cyst
ED: Emergency department
GI: Gastrointestinal
LI-RADS v2018: Liver Imaging Reporting and Data System Version 2018
LM: Lymphatic malformation
MCDK: Multicystic dysplastic kidney
MEN1: Multiple endocrine neoplasia type I
MR: Magnetic resonance
MRCP: Magnetic resonance cholangiopancreatography
NET: Neuroendocrine tumor
PKD: Polycystic kidney disease
PLD: Polycystic liver disease
RCC: Renal cell cancer
T1W: T1-weighted
T2W: T2-weighted
TSC: Tuberous sclerosis complex
US: Ultrasonography
VHL: von Hippel–Lindau
VMC: Von Meyenburg complex
